# Penetration of Hydrogen into Polymer Electrolyte Membrane for Fuel Cells by Quantum and Molecular Dynamics Simulations

**DOI:** 10.3390/polym13060947

**Published:** 2021-03-19

**Authors:** JinHyeok Cha, Wooju Lee, Jihye Baek

**Affiliations:** Institute of Fundamental and Advanced Technology, Hyundai Motor Company, 37 Cheoldobangmulgwan-ro, Uiwang-si 16082, Gyeonggi-do, Korea; wooju@hyundai.com (W.L.); jh.baek@hyundai.com (J.B.)

**Keywords:** polymer electrolyte membrane for fuel cell, molecular dynamics simulations, side chain, penetration

## Abstract

The advent of the Hydrogen Society created great interest around hydrogen-based energy a decade ago, with several types of vehicles based on hydrogen fuel cells already being produced in the automotive sector. For highly efficient fuel cell systems, the control of hydrogen inside a polymer-based electrolyte membrane is crucial. In this study, we investigated the molecular behavior of hydrogen inside a polymer-based proton-exchange membrane, using quantum and molecular dynamics simulations. In particular, this study focused on the structural difference of the pendent-like side chain polymer, resulting in the penetration ratio of hydrogen into the membrane deriving from the penetration depth of the membrane’s thickness while keeping the simulation time constant. The results reveal that the penetration ratio of the polymer with a shorter side chain was higher than that with the longer side chain. This was justified via two perspectives; electrostatic and van der Waals molecular interactions, and the structural difference of the polymers resulting in the free volume and different behavior of the side chain. In conclusion, we found that a longer side chain is more trembling and acts as an obstruction, dominating the penetration of hydrogen inside the polymer membrane.

## 1. Introduction

Hydrogen, as an energy resource, can make life significantly more eco-friendly. Although the hydrogen fuel cell was introduced by William Robert Grove 180 years ago [1], numerous potential applications have recently raised extreme engagement from many researchers, in various industrial sectors [2]. Automotive systems based on hydrogen fuel cells can already be found on the road, with examples such as “NEXO” produced by Hyundai Motor Company or “MIRAI” by Toyota representing the upcoming hydrogen-based automotive future.

A hydrogen fuel cell works based on electrochemistry, by passing hydrogen through the anode and oxygen through the cathode of the cell. More specifically, electrical power is generated via three simple steps: (i) the dissociation of hydrogen to the proton and electron; (ii) the conduction of electrons through electronic channels and protons through a proton-exchange membrane; and (iii) the synthesis of water by a proton, electron, and oxygen. A polymer electrolyte membrane for fuel cell (PEMFC) is commonly used in vehicles due to better operation at relatively low temperature, while other types of fuel cells (such as alkaline cell, phosphoric acid fuel cell, molten carbonate cell, direct methanol fuel cell and solid oxide cell) require a higher operation temperature [3,4].

**Figure 1:**
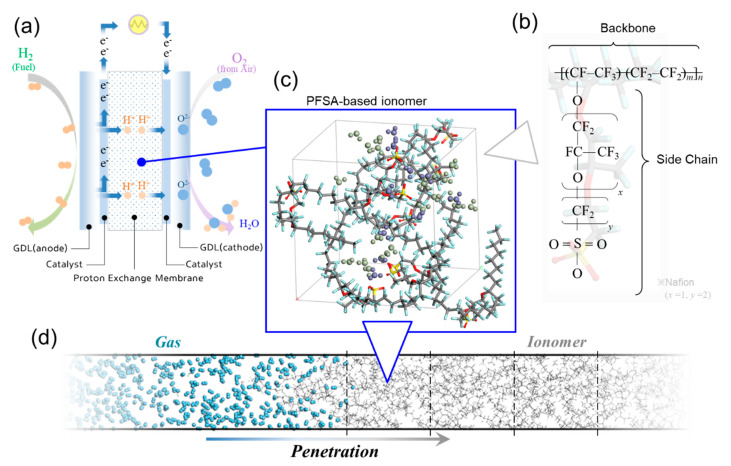
(**a**) Schematic of the operation of a fuel cell; (**b**) snapshot of the unit cell for the molecular dynamic (MD) simulation of the morphological effect of the side chain on gas penetration, containing H_3_O^+^ and H_2_O with the hydration number of 3; (**c**) index *x* (backbone) and *y* (side chain) of the chemical structure of perfluorosulfonic acid determine the length of side chain; and (**d**) schematic of the MD simulation for hydrogen molecules’ penetration into the ionomer region.

A stack, one of the main components of PEMFCs, comprises of serially connected unit cells generating electricity. Each of them features a bipolar gas diffusion layer (GDL) and a membrane electrode assembly (MEA), which are directly responsible for the electrochemical reaction. Figure 1a shows a schematic of a proton exchange membrane in the fuel cell system. The proton exchange membrane is a key component of PEMFCs, acting excellently towards gas blocking and proton conduction. In particular, gas diffusion behavior inside MEA has a direct impact on the performance of the fuel cell, as the diffusion of hydrogen and oxygen at both the anode and cathode determines the efficiency of the fuel cell, hence shortened diffusion time induces faster proton transport and water conversion [5,6]. In addition, gas crossover through the PEMFC during long-term membrane operation causes the degradation of the MEA, due to the combustion reaction between H_2_ and O_2_ gases and the formation of reactive oxygen radicals (HO• and HOO• radicals) which attack membrane linkages [7,8,9,10,11].

Gas is transported through carriers of polymeric surrounding, such as a polymer-based binder supporting catalysts at both electrodes, and a polymeric electrolyte membrane. Therefore, the interaction between the gas molecules and polymer needs to be clarified, in order to control the gas flow in and out. The transport of gas molecules in the PEM depends on the geometric properties of polymeric media as well as atomic interactions between gas and polymer molecules. The geometrical effects of polymeric media can be analyzed by using a theoretical model of diffusion in porous media. Several studies have been reported on the diffusion phenomena in the randomly distributed microstructure by employing fractal geometry theory [12,13], and those studies show a good agreement with the available experimental data and existing models reported in the literature. Even the theoretical model of diffusion in the porous media well predicts the effective transport properties of gas molecules, the effect of molecular interactions should be fully understood to design polymeric materials in molecular-level (e.g., chain length, electrostatic interaction, radius of gyration of polymer formed by different chain structures).

To explore the molecular behavior of gas (e.g., hydrogen at the anode and oxygen at the cathode) [14], numerous studies have employed molecular dynamic (MD) simulations and first-principles simulations [15,16,17]. MD simulation is a method of calculating and statistically processing material structures, thermodynamic properties, and reaction properties at atomic scales. It mathematically solves the kinetic trajectory of atoms and molecules based on Newton’s equations of motion for the hundreds and hundreds of thousands of molecules that change over time by parametrizing the interaction potential between atoms. Kwon et al. evaluated the molecular behavior inside the polymeric membrane formed with a different equivalent weight (EW) of side chains at various hydrated levels by volume analysis that the lower EW of the ionomer exhibits higher diffusion coefficients of particles such as water, hydronium, and oxygen molecules [15]. Cha explored the morphological effect of the side chain on H_3_O^+^ transfer that short side chains induced more inter-chain cation movement than longer side chains [16]. Takeuchi et al. investigated that the local crystalline structure which inhibits the H_2_ diffusion across the aligned polymer chains due to the void fraction in the structure, resulting in reducing H_2_ permeability [17]. Jinnouchi et al. reported the transport properties of O_2_ molecules inside Nafion ionomer using the density functional theory (DFT) calculation and MD simulations, showing that interfacial formation between Nafion and Pt significantly enhances O_2_ permeation through the ionomer thin film [18]. Although all of these studies mainly used MD simulation to investigate the phenomena for molecular behavior in the polymeric membrane, however, there is still a lack of a more detailed understanding of the correlations between the structural characteristics of the polymer and the penetration of molecular behavior. To facilitate the research on polymer characteristics, it is necessary to focus on the interface between polymer and gas molecules, exploring the effect of properties of the polymer such as its type, structure, and ability for penetration by a specific gas.

In this study, we performed MD simulations focusing on hydrogen penetration into a polymer matrix. We employed a quantitative evaluation method, examining the effect of morphological differences of the polymer matrix on the penetration depth achieved. This study used two perspectives; molecular interaction (such as electrostatic and van der Waals interaction) and structural difference resulting in the free volume and behavior of polymer side chains. The results reveal that membranes based on polymer containing shorter side chains led to a higher penetration ratio than that of longer side chains. In addition, the increased trembling of the longer side chain compared to the shorter one acted as an obstruction, dominating the penetration ability of hydrogen inside the polymer.

## 2. Simulation Methodology

### 2.1. Models of Polymer Electrolyte Membranes and Hydrogen

A crucial factor of designing the MEA is that hydrogen from GDL should not penetrate into the polymer electrolyte membrane, resulting in the efficiency of the fuel cell system. Since the main purpose of this study is to evaluate the undesirable diffusion of undissociated hydrogen into the membrane, the dissociation process of hydrogen at the Pt particles was neglected. In other words, all of the hydrogen molecules penetrating the membrane were considered to be crossover. Based on this assumption, we only investigated the structural effects of the polymer on hydrogen penetration into the membrane.

We employed MD simulations to clarify the structural and kinetic properties of the polymer, which can calculate material structures, thermodynamic properties, and reaction properties at atomic scales. However, the MD simulation is hard to describe the varying hydration level and temperature of the real PEMFC system due to the large difference in time-scale between the simulation (ns) and operation (s). To simplify the calculation, we assumed that the hydration level and temperature were fixed. Several studies have reported selective penetration techniques under development, such as prevention strategies for the crossover of hydrogen from anode side, and the oxygen from cathode [14,19]. To investigate the effect of polymer structure on hydrogen penetration into the polymer electrolyte membrane, we employed perfluorosulfonic acid (PFSA)-based ionomer (commercially known as Nafion, most commonly used in fuel cells developed by DuPont Inc. more than 40 years ago) [20]. Figure 1b show the chemical structure of the polymer, including a backbone (–CF_2_–) and pendent–like side chain (–O[CF_2_CF(CF_3_)O]*_x_*[CF_2_]*_y_*SO_3_–) groups (index *x* and *y* of Nafion is equal to 1 and 2, respectively). The equivalent weight of the Nafion employed in this study was 1.147.

**Figure 2:**
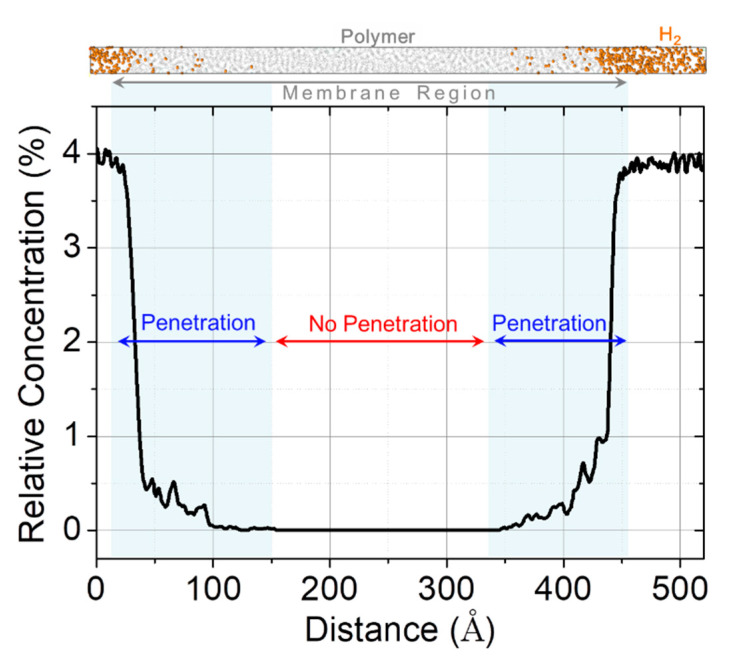
Relative concentration profile of hydrogen molecules. The simulation system is divided into penetrated and not penetrated regions. Their ratio is considered as a criterion for the comparison of penetration as a result of the side chain morphology.

To determine the structural difference of the side chain as longer and shorter, we varied “*y*” whilst keeping “*x*” constant. The structural length of the side chain is considered an impactful factor for the penetration of hydrogen at the surface of the polymer electrolyte membrane. The chain length ranged from 4.66 to 5.98 Å, and the number of atoms in a single chain was 582, 682, 982, and 1282, corresponding to *y* values of 1, 2, 5 and 8, respectively. The unit cell contained a single PFSA chain with 10 side chains, indicating 10 negatively charged sulfonate groups. Therefore, we added 10 positively charged H_3_O^+^ groups to the unit cell, to maintain the equivalent net charge constant. Moreover, each unit cell featured 20 H_2_O molecules, as the hydration number (*λ*) was set to be 3, as shown in Figure 2c. The simulation system composed of 20 unit cells with adjusted density to about 1.7 g/cm^3^, considering the hydration state [21]. The simulation system consisted of a rectangular cell with length ranging from 2.14 × 2.14 × 42.9 nm to 2.41 × 2.41 × 48.3 nm for the polymer membrane, and 2.30 × 2.30 × 6.33 nm for the hydrogen layer, resulting in a volume of 197.0–281.2 nm^3^ and 33.5 nm^3^, respectively. The density of the cell for hydrogen layer was kept constant at 0.05 g/cm^3^, corresponding to ≈70 MPa, equivalent to the pressure in hydrogen storage tank.

### 2.2. Simulation Details

All MD simulations were based on the condensed-phase optimized molecular potentials for atomistic simulation studies (COMPASS) force field potential in the software package Materials Studio 2016 (BIOVIA Software Inc., San Diego, CA, USA), which is a general all-atom force field for the atomistic simulation of common organic molecules, inorganic small molecules, and polymers [22,23].

The Verlet velocity algorithm was used for the integration of the motion equations [24]. Simulations were performed at room temperature (298 K), and regulated using a Nose–Hoover–Langevin thermostat [25,26]. The *NVT* ensemble was employed to identify hydrogen molecular behavior with a 1 fs time step, where *N*, *V* and *T* correspond to the number of atoms, volume, and temperature of the simulation system, respectively. We carried out each simulation for 1 ns and repeated it 10–15 times for each different initial structure, excluding data from the initial 200 ps assuming unexpected molecular behavior.

Density functional theory (DFT) calculations were carried out to clarify the electrostatic interaction between hydrogen and the side chain using the Dmol^3^ program, which is a theory for computing the shape of electrons within a molecule, and their energy based on quantum mechanics. This is one of the most widely used quantum mechanics calculations that allows to predict whether the molecule can exist in the world or not, and the form and properties of a particular molecule. Through the charge distribution of molecules calculated by DFT, it enabled accurately simulating the behavior of particles by representing the electrostatic interaction (Coulomb interaction) between molecules. The electronic exchange-correlation functional used was the Perdew–Burke–Emzerhog (PBE) functional with the generalized gradient approximation (GGA) [27], and the spin-polarized calculations were performed using a double numerical basis set with polarization functions (DNP). All electron relativistic effects were included for the treatment of core electrons in the models. The molecular binding energy of H_3_O^+^ or H_2_ on the side chain was determined as follows:

*E*_binding energy_ = *E*_total energy_ − *E*_side chain_ − *E*_molecules_ (1)

where *E*_total energy_ is the total energy of the side chain with the bound molecules, and the *E*_side chain_ and *E*_molecules_ are the total energies of the side chain and the molecules, respectively. Note that in order to calculate the binding energy of H_2_ on the side chain, the total energy includes the bonding state of H_3_O^+^ on side chain.

## 3. Results and Discussion

### 3.1. Quantitative Evaluation Model for the Effect of Side Chain on Penetration

There are unpredictably numerous polymeric structures for a given system, resulting from polymeric gyration, steric hindrance, and the free volume inside a system. However, it is practically impossible to explore the permeation of hydrogen into the polymer-based membrane for every possible structure. Thus, in this study, we focused on the structural effect of the side chain on hydrogen permeation, aiming to improve the design of polymer electrolyte membranes.

The concept of permeation implies that molecules pass through the membrane from the one side to the other. For most polymeric membranes, gas permeability is calculated by multiplying the diffusivity and solubility of the penetrant gas in the polymer [28]. However, it is very hard to observe the whole infiltration process from the one to the other side at once, because it requires an insurmountably long time and large ability to simulate it. MD simulation can describe a partial molecular behavior, for example on the membrane interface, focusing on the initial “penetration” of hydrogen into PFSA-based electrolyte membrane.

In this study, we investigated the instant interfacial phenomenon of molecular penetration from the hydrogen layer into the PFSA-based membrane using MD simulation, which can potentially provide physical and chemical information for the design of a hydrogen-controllable polymer for PEMFC fabrication. Figure 1d shows a snapshot of the MD simulation system, composed of a polymer region sufficiently long in the axial direction, and showing the combination of hydrogen and polymer layers during penetration. Then, we quantitatively evaluated the depth of hydrogen penetration into the polymer. The depth depends on the simulation time corresponding to solubility. The longer simulation time facilitates deeper hydrogen penetration into the polymer. However, the normalized depth ratio can overcome the dependence on constant simulation time, focusing on the structural effect of the polymer and its effect on hydrogen penetration.

Figure 2 shows the evaluation method employed to interpret results. First, we plotted the relative concentration profile of hydrogen in polymer over the axial direction for the entire simulation system. Then, we divided the polymer layer into two parts; the penetrated and the unpenetrated. The ratio of the length of membrane to penetration depth by hydrogen molecules ranged from 0 to 1, using normalized values as criteria for the comparison of penetration resulting from the morphology of the side chain. Whilst “0” meant that membrane was not at all penetrated, “1” implied full hydrogen penetration.

**Figure 3:**
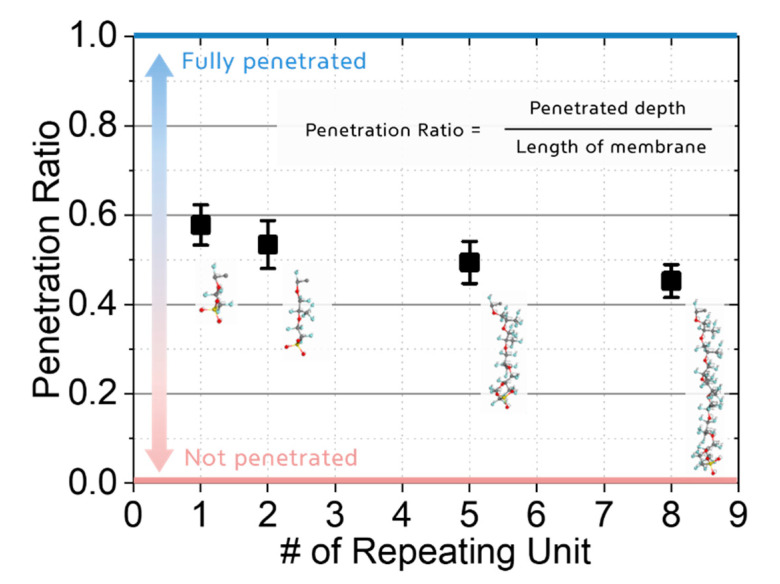
The effect of the side chain length on the penetration depth into polymeric membrane. The longer side chain (8 repeating units) causes hydrogen molecules to penetrate 21.7% deeper compared to the penetration achieved with the shortest side chain (1 repeating unit).

Figure 3 shows the effect of the side chain on hydrogen penetration into the polymer membrane. A shorter side chain allowed hydrogen to penetrate deeper inside the membrane compared to a longer chain. In particular, the PFSA-based polymer with the longest side chain in this study (eight repeating units) allowed for a 21.7% deeper hydrogen penetration compared to that with the shortest chain (one repeating unit). We devised three potential reasons to explain the obtained results. We assumed that the interaction caused from the electrostatic Coulomb force or van der Waals interaction between the hydrogen and each side chain resulted in hydrogen penetration. Structural differences of each side chain could also lead to a different free volume inside the system and accelerate or decelerate the molecular transport of hydrogen. Moreover, the different physical behavior of the side chain resulting from its length could facilitate or disturb hydrogen penetration inside the polymer membrane.

### 3.2. Hydrogen Adsorption onto the Side Chain

**Figure 4:**
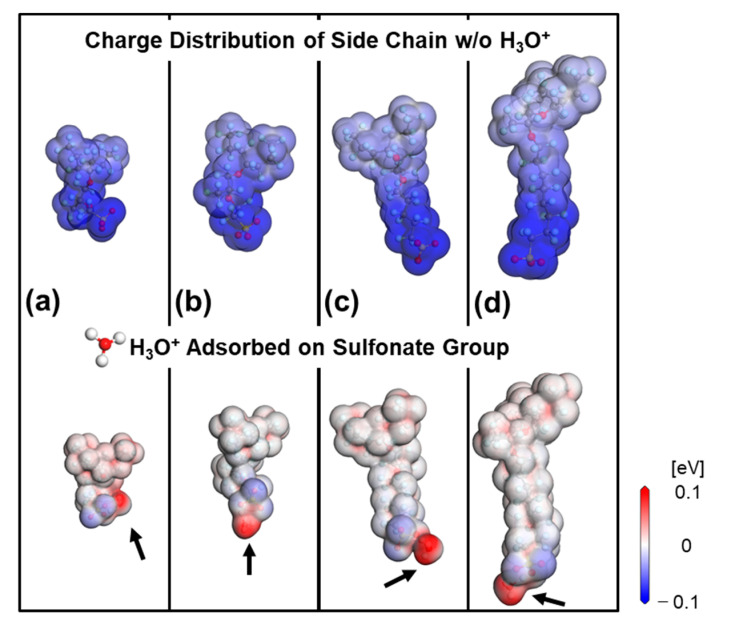
Charge distribution of the side chain before and after H_3_O^+^ is adsorbed on the negatively charged sulfonate group. H_3_O^+^adsorption leads to neutralization despite the chain length. The length index *y* corresponds to each of the (**a**)–(**d**) side chain is 1, 2, 5 and 8, respectively.

Hydrogen provided by the gas diffusion layer is dissociated with electron and proton with the aid of the catalyst. As described above, a membrane with selective permeability for proton and hydrogen is an essential factor for the fuel cell efficiency. Hydrogen interacts with Nafion electrostatically, and Nafion’s polymeric structure contains a hydrophobic backbone (−CF_2_−) and a hydrophilic side chain having a negatively charged sulfonate group (−SO_3_^−^). This implies that the longer the length of a side chain is, the greater the difference in the charge distribution within the side chain will be. Therefore, we investigated the interaction of hydrogen with a side chain, having a negatively charged sulfonate group at the end, using DFT calculations. Figure 4 shows the comparison of the charge distribution for the side chains of various lengths, with or without H_3_O^+^ attached on the sulfonate group, corresponding to the lower and upper part of the figure respectively. The sulfonate group exhibits strong attractive interaction, leading to a hydrophilic network with H_3_O^+^. Negatively charged distribution along the side chain was neutralized by the attached H_3_O^+^ onto sulfonate group, irrespectively of the chain length. Consequently, we concluded that the electrostatic interaction between hydrogen and the side chain was weak enough to be considered negligible.

**Figure 5:**
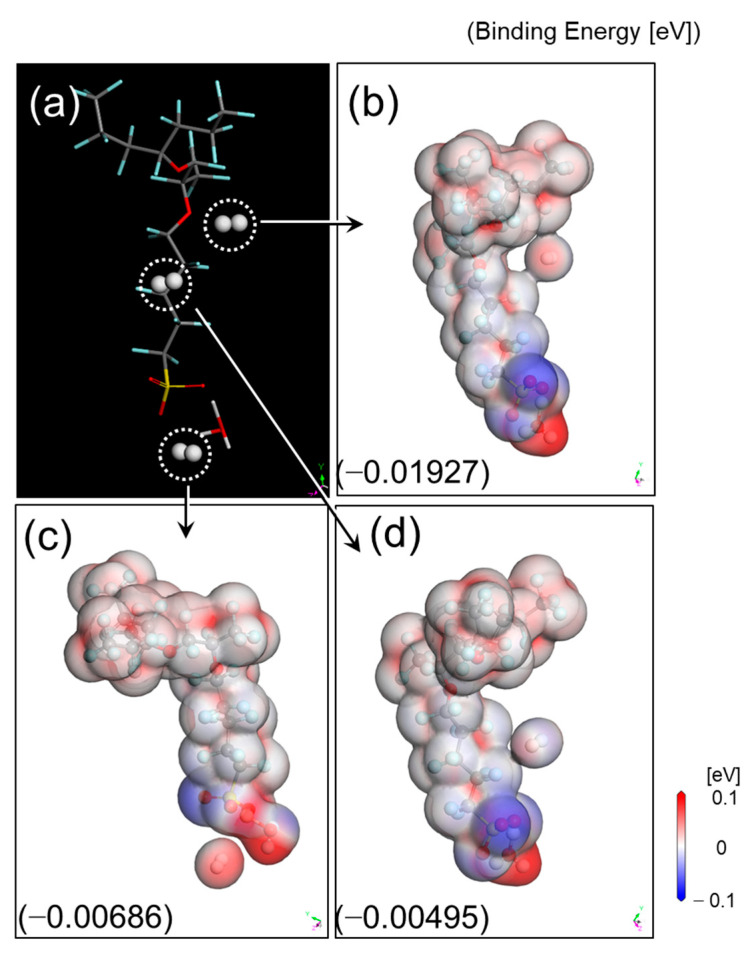
(**a**) Binding energy of hydrogen molecules on side chain with adsorbed H_3_O^+^; (**b**) possible positions for the adsorption of H_2_ molecules; (**c**) charge distribution; and (**d**) binding energy.

On the other hand, we considered the binding energy resulting from van der Waals forces driven by induced electrical interactions between two close atoms. The three positions selected to calculate binding energy are as shown in Figure 5a, close to the backbone, middle of the side chain, and around the adsorption of the sulfonated group and hydronium. The binding energy of the hydrogen molecules on side chain with adsorbed H_3_O^+^ ranged from −0.005 to −0.02 eV (Figure 5b–d), obviously referring to physical adsorption between them, with the distance ranging from 3.0 to 3.5 Å Therefore, we can safely conclude that van der Waals interaction has a negligible effect on hydrogen molecular behavior into the polymeric membrane, although the longer side chain offers a wider surface for physisorption.

### 3.3. Physical Obstruction against the Molecular Penetration

**Figure 6:**
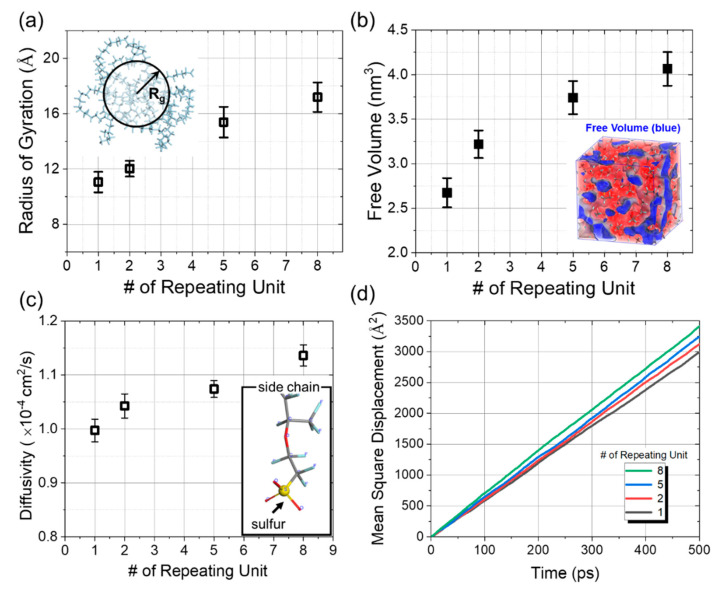
(**a**) Increase in the radius of gyration with the repeating unit number, which has a physical influence on the external molecular behavior; (**b**) the free volume with the repeating unit number for each simulation system, presenting the length of side chain; and (**c**) the increase in the diffusivity of the sulfur atom at the end of the side chain length, derived from (**d**) the mean square displacement increasing with the number of repeating units.

Hydrogen molecules are chemically stable without any reaction during the simulation, and DFT simulations also found only negligible chemical interactions through the binding energy calculations described in Section 3.2. Therefore, we focused on physical factors which can be divided into two categories: structural and kinetic. There are several representative polymer structures, such as linear, branched, cross-linked, and networked. Polymers containing side chain in particular, can be described by numerous complex morphologies. Thus, it is unfeasible to examine all these structures for their morphological influence, so we only varied the morphology of the side chain that causes hydrogen penetration, which is one of the most factors under the same composition. We found the effect of structure based on a single chain containing various side chain on hydrogen penetration. For that, we employed the radius of gyration (*R*_g_), a basic concept for the feature of polymer structures, and free volume formed from it. For that, we employed the *R*_g_, a basic concept for the feature of polymer structures, defined as the root-mean-square (RMS) distance of the collection of atoms in the molecule from their common center of mass, and calculated from the following Equation:

(2) $$R_{g}{}^{2}=\sum_{i=1}^{N}m_{i}s_{i}{}^{2}/\sum_{i=1}^{N}m_{i}$$

where *s_i_*, *m_i_* and *N* denote the distance of atom *i* from the center of mass, the mass of atom *i*, and the total number of atoms, respectively. Figure 6a shows the dependence of *R*_g_ on the side chain length that the *R*_g_ of the longest longer side chain is 1. 5 times larger than that of the shortest. Since the difluoromethylene group tends to aggregate in water due to the hydrophobicity of the backbone, the longer side chain with a stronger hydrophilic group has higher *R*_g_ than shorter side chains. The increase in *R*_g_ resulting from the longer side chain led to the simulation system containing more free volume, since the system needed to adjust the volume in order to keep the density constant, as shown Figure 6b. The free volume measured for the longest side chain employed in this study was 1.5 times larger than that of the shortest. Although having more free volume could lead to increased hydrogen penetration, the simulation results contradicted that. The simulation was carried out for the instant phenomenon at the interface, which was not enough to thoroughly investigate the effect of free volume on hydrogen penetration, due to the low density (or pressure) inside the polymer layer where hydrogen behaves. For further clarification of the influence of free volume, the model should be built in a fully solvated state.

In addition to morphology, we also investigated the effect of the side chain on the polymer’s kinetic behavior. More specifically, to find the kinetic behavior of the side chain, we calculated the molecular diffusivity, often called the diffusion coefficient (*D*), using Equation (3):

(3) $$D=\frac{1}{6}\underset{\Delta t\to \infty }{lim}\frac{dMSD}{d\Delta t}$$

where *MSD*, the mean square displacement of sulfur at the end of side chain, is given by

(4) $$MSD\equiv \langle {\left(x-x_{0}\right)}^{2}\rangle =\frac{1}{T}\sum_{t=1}^{T}{\left(x(t)-x_{0}\right)}^{2}$$

where *T* is the average time and *x*_0_ is the reference position of the particle. We found that an increased number of repeating units leads to the high diffusivity of the sulfur atom. Figure 6c shows the diffusivity of the sulfur atom at the end of side chains of various lengths, derived from the mean square displacement that increased with the number of repeating units, as shown in Figure 6d. Since the sulfur atoms are bonded at the end of the side chain, the diffusion of sulfur atoms presented in this study does not imply diffusion into other spaces, but rather the degree of movement within the space allowed by the side chain. Considering the analysis of diffusion coefficient, we believe that the more kinetic behavior of longer side chains mainly acts as a hindrance to hydration molecules penetration into the polymer membrane.

The operating temperature of PEMFC reached up to 65 °C decreases the activation loss of the catalyst and increases voltage loss (ohmic loss) and hydrogen crossover, and changes relatively to humidity to be lower than at room temperature. The MD simulation with the *NVT* ensemble employed in this study is unable to present the change in relative humidity, including the phase transition of water at every moment. Thus, we performed the simulations at room temperature where those changes do not occur. Since the temperature affects the behavior of hydrogen more than that of the polymer, we thus expect that higher temperature allows relatively more hydrogen penetration. In the near future, we carried out more simulations with various conditions to break through the limiting factors found in this study.

## 4. Conclusions

In summary, over the past decade, numerous studies have been reported on hydrogen-based energy systems, targeting industrial applications. Several types of vehicles based on hydrogen fuel cells have already been produced. For the fuel cell system to be efficient, it is crucial to control the hydrogen inside the polymer-based electrolyte membrane which transfers it, without its dissociation into protons and electrons, as it decreases the fuel efficiency. Thus, it is necessary to clarify the penetration mechanism of hydrogen on the surface of the polymer, and the effect of the polymer properties, such as the type, structure, and size on penetrations. In this study, we used molecular dynamics simulations to investigate the mechanism of hydrogen molecular behavior inside a polymer-based proton exchange membrane. Using a quantitative evaluation method and focusing on how deeply hydrogen penetrates into morphologically different polymers, this study was approached through two perspectives. The first was molecular interactions, such as electrostatic and van der Waals interactions, and the second was the structural difference, resulting in differences in free volume and the behavior of side chain. The results show that the polymer membrane containing a shorter side chain caused a higher penetration ratio than a longer side chain. In addition, the more trembling longer side chain acted as an obstruction, dominating the penetration of hydrogen inside the polymer. With the results obtained in this study as the beginning point, we further expanded the research to find the effect of the hydration level, different temperature, the addition of additives, and a hybrid membrane with a skeleton considering the improvement of durability, on hydrogen crossover, which contributes to estimating the optimal design factors for a polymer-based electrolyte membrane.

## Data Availability

The data presented in this study are available on request from the corresponding author.
